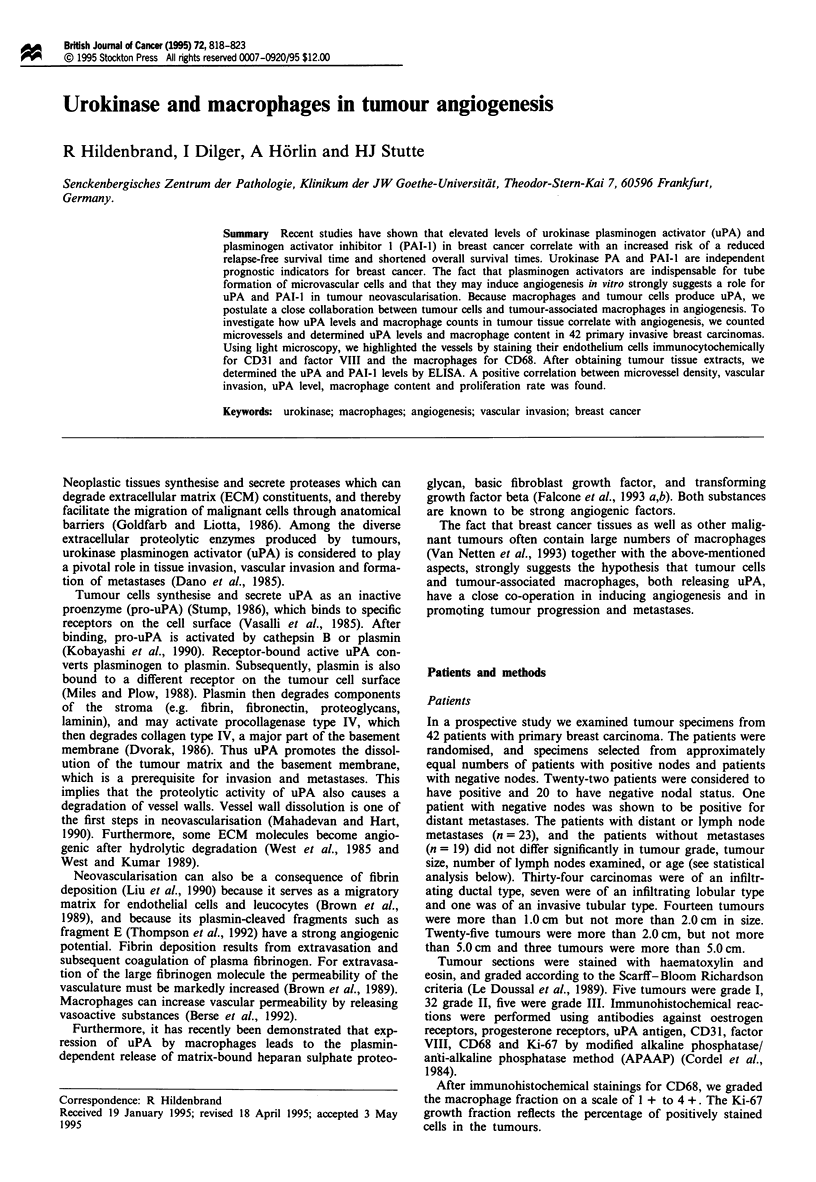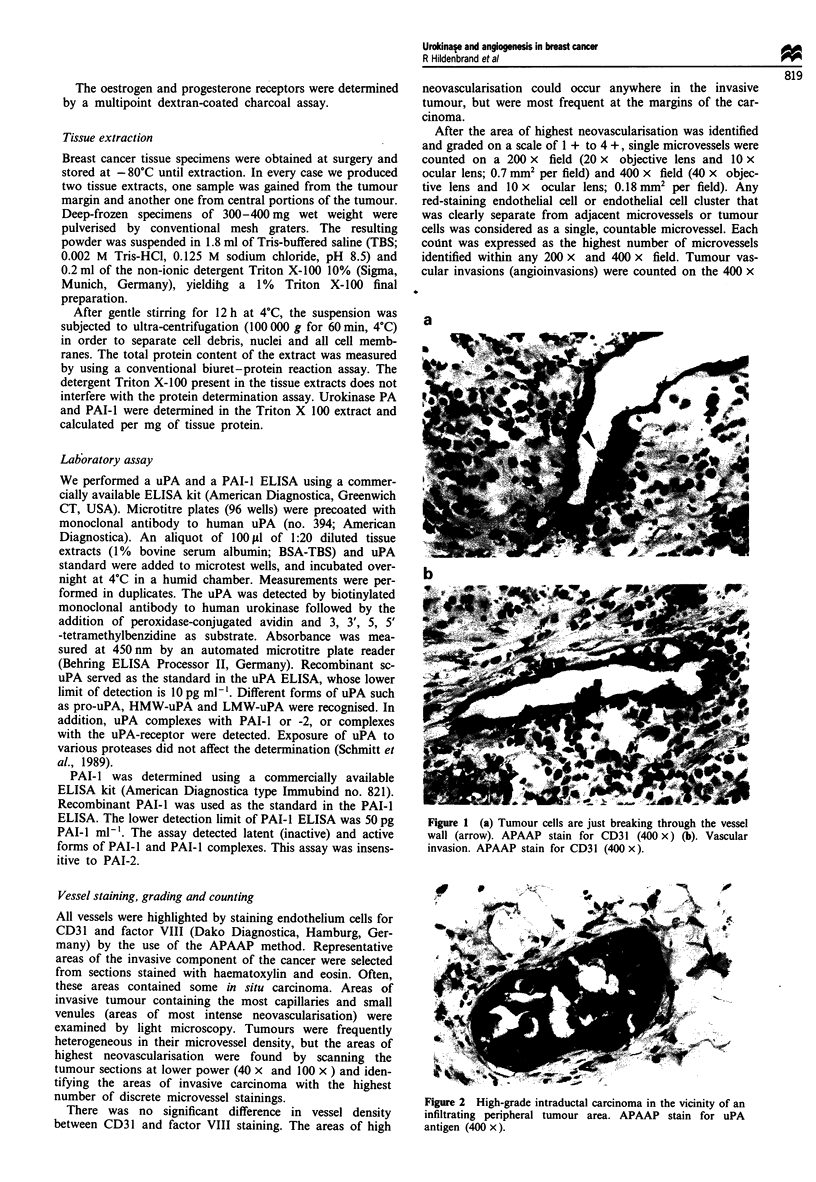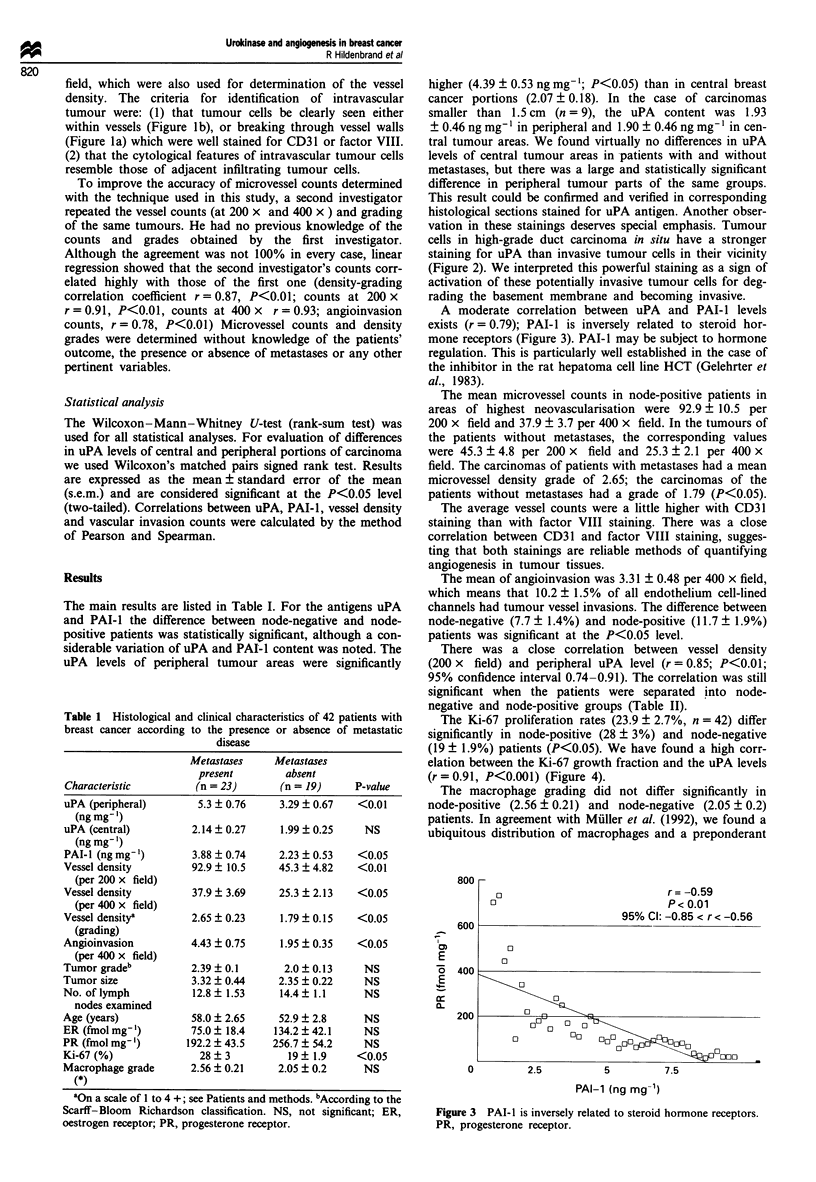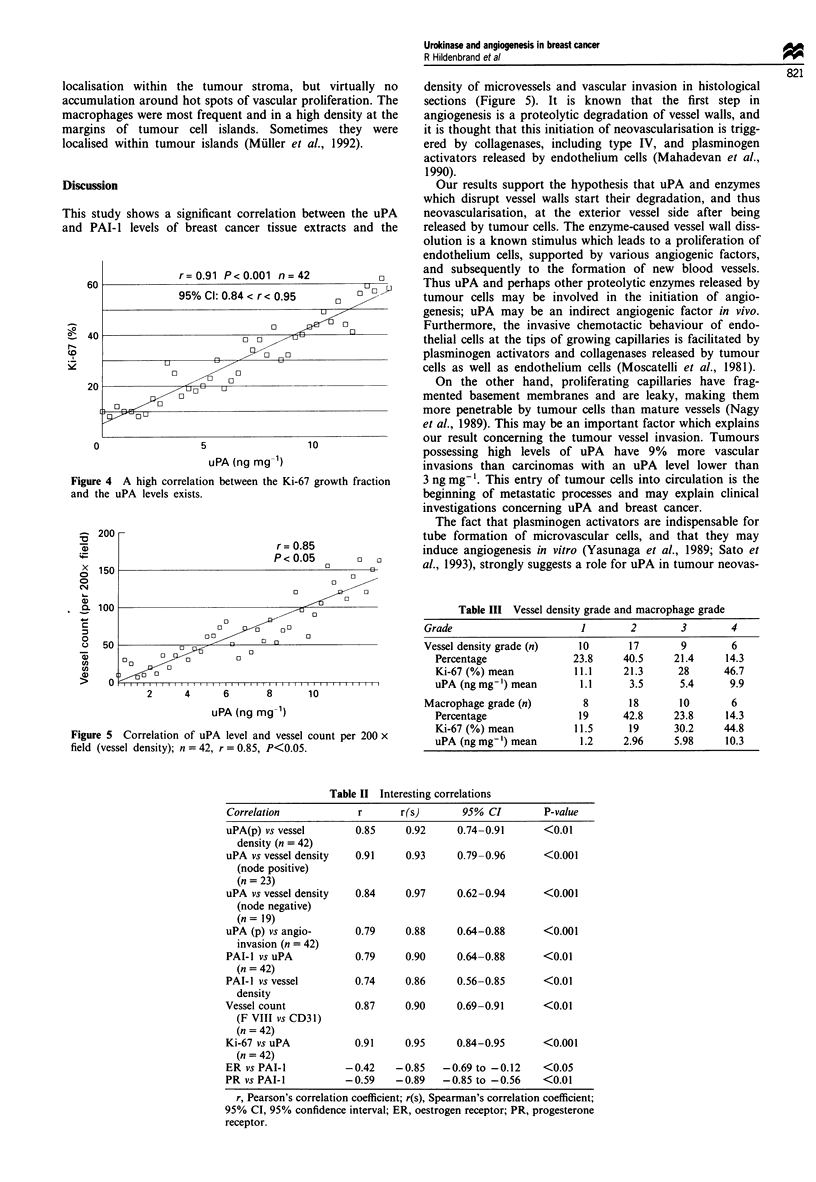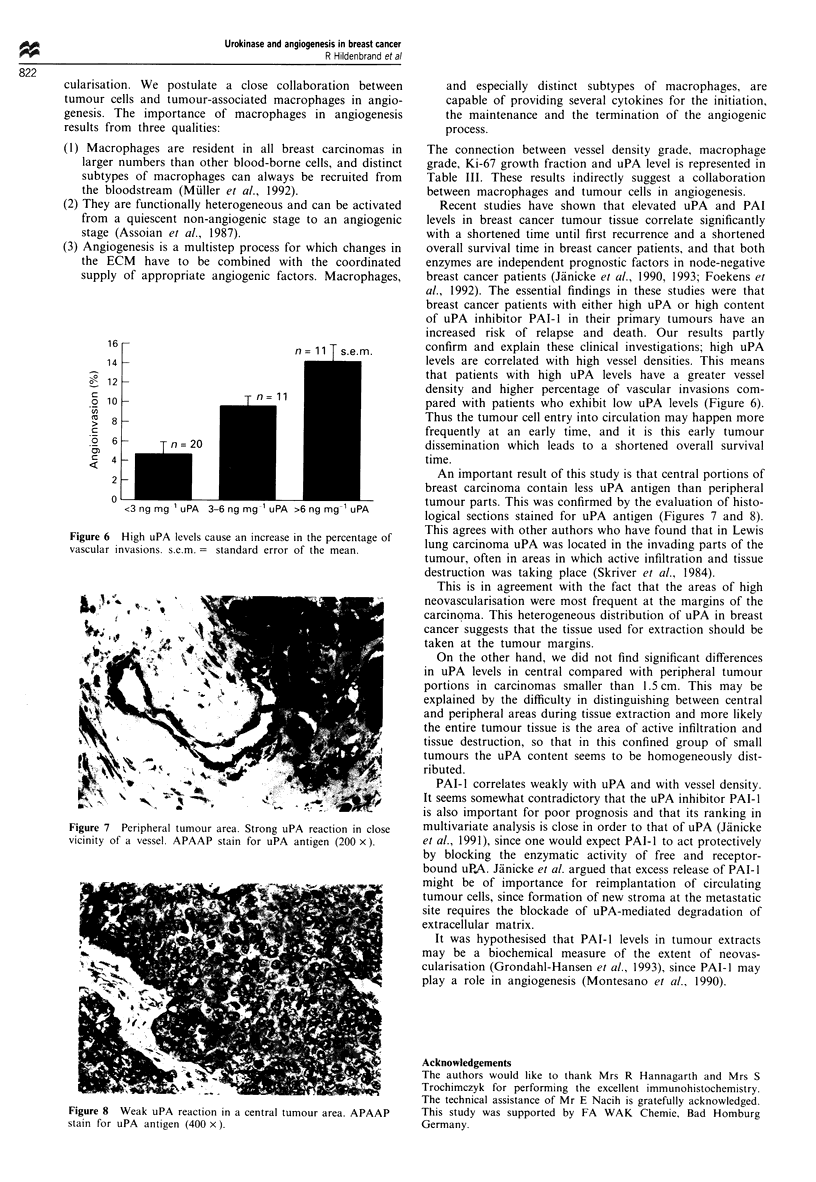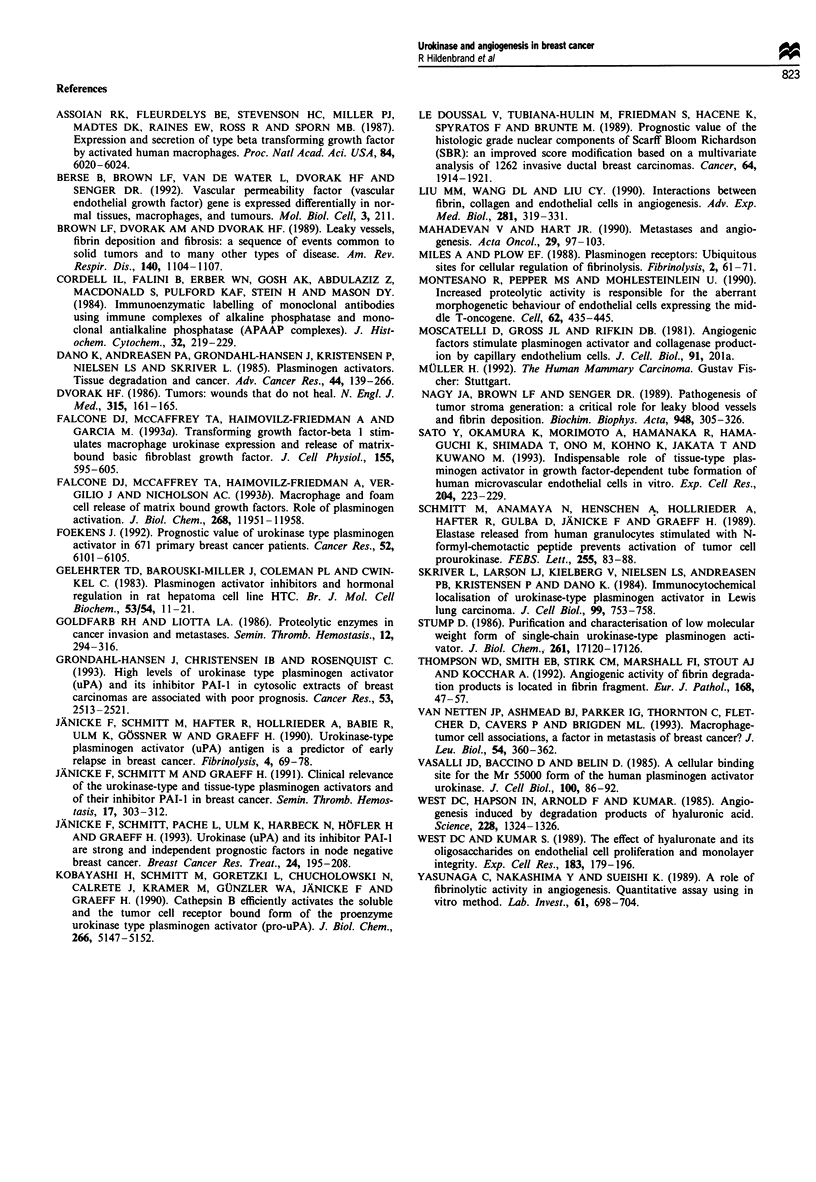# Urokinase and macrophages in tumour angiogenesis.

**DOI:** 10.1038/bjc.1995.419

**Published:** 1995-10

**Authors:** R. Hildenbrand, I. Dilger, A. Hörlin, H. J. Stutte

**Affiliations:** Senckenbergisches Zentrum der Pathologie, Klinikum der JW Goethe-Universität, Frankfurt, Germany.

## Abstract

**Images:**


British Journal of Cancer (1995) 72, 818-823
© 1995 Stockton Press All rights reserved 0007-0920/95 $12.00

### Urokinase and macrophages in tumour angiogenesis

R Hildenbrand, I Dilger, A Hörlin and HJ Stutte

*Senckenbergisches Zentrum der Pathologie, Klinikum der JW Goethe-Universität, Theodor-Stern-Kai 7, 60596 Frankfurt, Germany.*

**Summary** Recent studies have shown that elevated levels of urokinase plasminogen activator (uPA) and plasminogen activator inhibitor 1 (PAI-1) in breast cancer correlate with an increased risk of a reduced relapse-free survival time and shortened overall survival times. Urokinase PA and PAI-1 are independent prognostic indicators for breast cancer. The fact that plasminogen activators are indispensable for tube formation of microvascular cells and that they may induce angiogenesis *in vitro* strongly suggests a role for uPA and PAI-1 in tumour neovascularisation. Because macrophages and tumour cells produce uPA, we postulate a close collaboration between tumour cells and tumour-associated macrophages in angiogenesis. To investigate how uPA levels and macrophage counts in tumour tissue correlate with angiogenesis, we counted microvessels and determined uPA levels and macrophage content in 42 primary invasive breast carcinomas. Using light microscopy, we highlighted the vessels by staining their endothelium cells immunocytochemically for CD31 and factor VIII and the macrophages for CD68. After obtaining tumour tissue extracts, we determined the uPA and PAI-1 levels by ELISA. A positive correlation between microvessel density, vascular invasion, uPA level, macrophage content and proliferation rate was found.

**Keywords:** urokinase; macrophages; angiogenesis; vascular invasion; breast cancer

Neoplastic tissues synthesise and secrete proteases which can degrade extracellular matrix (ECM) constituents, and thereby facilitate the migration of malignant cells through anatomical barriers (Goldfarb and Liotta, 1986). Among the diverse extracellular proteolytic enzymes produced by tumours, urokinase plasminogen activator (uPA) is considered to play a pivotal role in tissue invasion, vascular invasion and formation of metastases (Dano *et al.*, 1985).

Tumour cells synthesise and secrete uPA as an inactive proenzyme (pro-uPA) (Stump, 1986), which binds to specific receptors on the cell surface (Vasalli *et al.*, 1985). After binding, pro-uPA is activated by cathepsin B or plasmin (Kobayashi *et al.*, 1990). Receptor-bound active uPA converts plasminogen to plasmin. Subsequently, plasmin is also bound to a different receptor on the tumour cell surface (Miles and Plow, 1988). Plasmin then degrades components of the stroma (e.g. fibrin, fibronectin, proteoglycans, laminin), and may activate procollagenase type IV, which then degrades collagen type IV, a major part of the basement membrane (Dvorak, 1986). Thus uPA promotes the dissolution of the tumour matrix and the basement membrane, which is a prerequisite for invasion and metastases. This implies that the proteolytic activity of uPA also causes a degradation of vessel walls. Vessel wall dissolution is one of the first steps in neovascularisation (Mahadevan and Hart, 1990). Furthermore, some ECM molecules become angiogenic after hydrolytic degradation (West *et al.*, 1985 and West and Kumar 1989).

Neovascularisation can also be a consequence of fibrin deposition (Liu *et al.*, 1990) because it serves as a migratory matrix for endothelial cells and leucocytes (Brown *et al.*, 1989), and because its plasmin-cleaved fragments such as fragment E (Thompson *et al.*, 1992) have a strong angiogenic potential. Fibrin deposition results from extravasation and subsequent coagulation of plasma fibrinogen. For extravasation of the large fibrinogen molecule the permeability of the vasculature must be markedly increased (Brown *et al.*, 1989). Macrophages can increase vascular permeability by releasing vasoactive substances (Berse *et al.*, 1992).

Furthermore, it has recently been demonstrated that expression of uPA by macrophages leads to the plasmin-dependent release of matrix-bound heparan sulphate proteoglycan, basic fibroblast growth factor, and transforming growth factor beta (Falcone *et al.*, 1993 *a,b*). Both substances are known to be strong angiogenic factors.

The fact that breast cancer tissues as well as other malignant tumours often contain large numbers of macrophages (Van Netten *et al.*, 1993) together with the above-mentioned aspects, strongly suggests the hypothesis that tumour cells and tumour-associated macrophages, both releasing uPA, have a close co-operation in inducing angiogenesis and in promoting tumour progression and metastases.

Correspondence: R Hildenbrand
Received 19 January 1995; revised 18 April 1995; accepted 3 May 1995

### Patients and methods

### *Patients*

In a prospective study we examined tumour specimens from 42 patients with primary breast carcinoma. The patients were randomised, and specimens selected from approximately equal numbers of patients with positive nodes and patients with negative nodes. Twenty-two patients were considered to have positive and 20 to have negative nodal status. One patient with negative nodes was shown to be positive for distant metastases. The patients with distant or lymph node metastases ($n = 23$), and the patients without metastases ($n = 19$) did not differ significantly in tumour grade, tumour size, number of lymph nodes examined, or age (see statistical analysis below). Thirty-four carcinomas were of an infiltrating ductal type, seven were of an infiltrating lobular type and one was of an invasive tubular type. Fourteen tumours were more than 1.0 cm but not more than 2.0 cm in size. Twenty-five tumours were more than 2.0 cm, but not more than 5.0 cm and three tumours were more than 5.0 cm.

Tumour sections were stained with haematoxylin and eosin, and graded according to the Scarff-Bloom Richardson criteria (Le Doussal *et al.*, 1989). Five tumours were grade I, 32 grade II, five were grade III. Immunohistochemical reactions were performed using antibodies against oestrogen receptors, progesterone receptors, uPA antigen, CD31, factor VIII, CD68 and Ki-67 by modified alkaline phosphatase/anti-alkaline phosphatase method (APAAP) (Cordel *et al.*, 1984).

After immunohistochemical stainings for CD68, we graded the macrophage fraction on a scale of 1 + to 4 +. The Ki-67 growth fraction reflects the percentage of positively stained cells in the tumours.

The oestrogen and progesterone receptors were determined by a multipoint dextran-coated charcoal assay.

*Tissue extraction*

Breast cancer tissue specimens were obtained at surgery and stored at −80°C until extraction. In every case we produced two tissue extracts, one sample was gained from the tumour margin and another one from central portions of the tumour. Deep-frozen specimens of 300–400 mg wet weight were pulverised by conventional mesh graters. The resulting powder was suspended in 1.8 ml of Tris-buffered saline (TBS; 0.002 M Tris-HCl, 0.125 M sodium chloride, pH 8.5) and 0.2 ml of the non-ionic detergent Triton X-100 10% (Sigma, Munich, Germany), yielding a 1% Triton X-100 final preparation.

After gentle stirring for 12 h at 4°C, the suspension was subjected to ultra-centrifugation (100 000 *g* for 60 min, 4°C) in order to separate cell debris, nuclei and all cell membranes. The total protein content of the extract was measured by using a conventional biuret–protein reaction assay. The detergent Triton X-100 present in the tissue extracts does not interfere with the protein determination assay. Urokinase PA and PAI-1 were determined in the Triton X 100 extract and calculated per mg of tissue protein.

*Laboratory assay*

We performed a uPA and a PAI-1 ELISA using a commercially available ELISA kit (American Diagnostica, Greenwich CT, USA). Microtitre plates (96 wells) were precoated with monoclonal antibody to human uPA (no. 394; American Diagnostica). An aliquot of 100 μl of 1:20 diluted tissue extracts (1% bovine serum albumin; BSA-TBS) and uPA standard were added to microtest wells, and incubated overnight at 4°C in a humid chamber. Measurements were performed in duplicates. The uPA was detected by biotinylated monoclonal antibody to human urokinase followed by the addition of peroxidase-conjugated avidin and 3, 3′, 5, 5′ -tetramethylbenzidine as substrate. Absorbance was measured at 450 nm by an automated microtitre plate reader (Behring ELISA Processor II, Germany). Recombinant sc-uPA served as the standard in the uPA ELISA, whose lower limit of detection is 10 pg $ml^{-1}$. Different forms of uPA such as pro-uPA, HMW-uPA and LMW-uPA were recognised. In addition, uPA complexes with PAI-1 or -2, or complexes with the uPA-receptor were detected. Exposure of uPA to various proteases did not affect the determination (Schmitt *et al.*, 1989).

PAI-1 was determined using a commercially available ELISA kit (American Diagnostica type Immubind no. 821). Recombinant PAI-1 was used as the standard in the PAI-1 ELISA. The lower detection limit of PAI-1 ELISA was 50 pg PAI-1 $ml^{-1}$. The assay detected latent (inactive) and active forms of PAI-1 and PAI-1 complexes. This assay was insensitive to PAI-2.

*Vessel staining, grading and counting*

All vessels were highlighted by staining endothelium cells for CD31 and factor VIII (Dako Diagnostica, Hamburg, Germany) by the use of the APAAP method. Representative areas of the invasive component of the cancer were selected from sections stained with haematoxylin and eosin. Often, these areas contained some *in situ* carcinoma. Areas of invasive tumour containing the most capillaries and small venules (areas of most intense neovascularisation) were examined by light microscopy. Tumours were frequently heterogeneous in their microvessel density, but the areas of highest neovascularisation were found by scanning the tumour sections at lower power (40 × and 100 × ) and identifying the areas of invasive carcinoma with the highest number of discrete microvessel stainings.

There was no significant difference in vessel density between CD31 and factor VIII staining. The areas of high neovascularisation could occur anywhere in the invasive tumour, but were most frequent at the margins of the carcinoma.

After the area of highest neovascularisation was identified and graded on a scale of 1 + to 4 +, single microvessels were counted on a 200 × field (20 × objective lens and 10 × ocular lens; 0.7 $mm^2$ per field) and 400 × field (40 × objective lens and 10 × ocular lens; 0.18 $mm^2$ per field). Any red-staining endothelial cell or endothelial cell cluster that was clearly separate from adjacent microvessels or tumour cells was considered as a single, countable microvessel. Each count was expressed as the highest number of microvessels identified within any 200 × and 400 × field. Tumour vascular invasions (angioinvasions) were counted on the 400 ×

**Figure 1** (**a**) Tumour cells are just breaking through the vessel wall (arrow). APAAP stain for CD31 (400 ×) (**b**). Vascular invasion. APAAP stain for CD31 (400 ×).

**Figure 2** High-grade intraductal carcinoma in the vicinity of an infiltrating peripheral tumour area. APAAP stain for uPA antigen (400 ×).

field, which were also used for determination of the vessel density. The criteria for identification of intravascular tumour were: (1) that tumour cells be clearly seen either within vessels (Figure 1b), or breaking through vessel walls (Figure 1a) which were well stained for CD31 or factor VIII. (2) that the cytological features of intravascular tumour cells resemble those of adjacent infiltrating tumour cells.

To improve the accuracy of microvessel counts determined with the technique used in this study, a second investigator repeated the vessel counts (at 200 × and 400 × ) and grading of the same tumours. He had no previous knowledge of the counts and grades obtained by the first investigator. Although the agreement was not 100% in every case, linear regression showed that the second investigator's counts correlated highly with those of the first one (density-grading correlation coefficient $r = 0.87$, $P<0.01$; counts at 200 × $r = 0.91$, $P<0.01$, counts at 400 × $r = 0.93$; angioinvasion counts, $r = 0.78$, $P<0.01$) Microvessel counts and density grades were determined without knowledge of the patients' outcome, the presence or absence of metastases or any other pertinent variables.

*Statistical analysis*

The Wilcoxon–Mann–Whitney *U*-test (rank-sum test) was used for all statistical analyses. For evaluation of differences in uPA levels of central and peripheral portions of carcinoma we used Wilcoxon's matched pairs signed rank test. Results are expressed as the mean ± standard error of the mean (s.e.m.) and are considered significant at the $P<0.05$ level (two-tailed). Correlations between uPA, PAI-1, vessel density and vascular invasion counts were calculated by the method of Pearson and Spearman.

### Results

The main results are listed in Table I. For the antigens uPA and PAI-1 the difference between node-negative and node-positive patients was statistically significant, although a considerable variation of uPA and PAI-1 content was noted. The uPA levels of peripheral tumour areas were significantly higher ($4.39 \pm 0.53$ ng mg$^{-1}$; $P<0.05$) than in central breast cancer portions ($2.07 \pm 0.18$). In the case of carcinomas smaller than 1.5 cm ($n = 9$), the uPA content was 1.93 ± 0.46 ng mg$^{-1}$ in peripheral and 1.90 ± 0.46 ng mg$^{-1}$ in central tumour areas. We found virtually no differences in uPA levels of central tumour areas in patients with and without metastases, but there was a large and statistically significant difference in peripheral tumour parts of the same groups. This result could be confirmed and verified in corresponding histological sections stained for uPA antigen. Another observation in these stainings deserves special emphasis. Tumour cells in high-grade duct carcinoma *in situ* have a stronger staining for uPA than invasive tumour cells in their vicinity (Figure 2). We interpreted this powerful staining as a sign of activation of these potentially invasive tumour cells for degrading the basement membrane and becoming invasive.

**Table 1** Histological and clinical characteristics of 42 patients with breast cancer according to the presence or absence of metastatic disease

| *Characteristic* | *Metastases present (n = 23)* | *Metastases absent (n = 19)* | P-*value* |
|---|---|---|---|
| uPA (peripheral) (ng mg$^{-1}$) | 5.3 ± 0.76 | 3.29 ± 0.67 | <0.01 |
| uPA (central) (ng mg$^{-1}$) | 2.14 ± 0.27 | 1.99 ± 0.25 | NS |
| PAI-1 (ng mg$^{-1}$) | 3.88 ± 0.74 | 2.23 ± 0.53 | <0.05 |
| Vessel density (per 200 × field) | 92.9 ± 10.5 | 45.3 ± 4.82 | <0.01 |
| Vessel density (per 400 × field) | 37.9 ± 3.69 | 25.3 ± 2.13 | <0.05 |
| Vessel density[a] (grading) | 2.65 ± 0.23 | 1.79 ± 0.15 | <0.05 |
| Angioinvasion (per 400 × field) | 4.43 ± 0.75 | 1.95 ± 0.35 | <0.05 |
| Tumor grade[b] | 2.39 ± 0.1 | 2.0 ± 0.13 | NS |
| Tumor size | 3.32 ± 0.44 | 2.35 ± 0.22 | NS |
| No. of lymph nodes examined | 12.8 ± 1.53 | 14.4 ± 1.1 | NS |
| Age (years) | 58.0 ± 2.65 | 52.9 ± 2.8 | NS |
| ER (fmol mg$^{-1}$) | 75.0 ± 18.4 | 134.2 ± 42.1 | NS |
| PR (fmol mg$^{-1}$) | 192.2 ± 43.5 | 256.7 ± 54.2 | NS |
| Ki-67 (%) | 28 ± 3 | 19 ± 1.9 | <0.05 |
| Macrophage grade (*) | 2.56 ± 0.21 | 2.05 ± 0.2 | NS |

[a]On a scale of 1 to 4 +; see Patients and methods. [b]According to the Scarff–Bloom Richardson classification. NS, not significant; ER, oestrogen receptor; PR, progesterone receptor.

A moderate correlation between uPA and PAI-1 levels exists ($r = 0.79$); PAI-1 is inversely related to steroid hormone receptors (Figure 3). PAI-1 may be subject to hormone regulation. This is particularly well established in the case of the inhibitor in the rat hepatoma cell line HCT (Gelehrter *et al.*, 1983).

The mean microvessel counts in node-positive patients in areas of highest neovascularisation were 92.9 ± 10.5 per 200 × field and 37.9 ± 3.7 per 400 × field. In the tumours of the patients without metastases, the corresponding values were 45.3 ± 4.8 per 200 × field and 25.3 ± 2.1 per 400 × field. The carcinomas of patients with metastases had a mean microvessel density grade of 2.65; the carcinomas of the patients without metastases had a grade of 1.79 ($P<0.05$).

The average vessel counts were a little higher with CD31 staining than with factor VIII staining. There was a close correlation between CD31 and factor VIII staining, suggesting that both stainings are reliable methods of quantifying angiogenesis in tumour tissues.

The mean of angioinvasion was 3.31 ± 0.48 per 400 × field, which means that 10.2 ± 1.5% of all endothelium cell-lined channels had tumour vessel invasions. The difference between node-negative (7.7 ± 1.4%) and node-positive (11.7 ± 1.9%) patients was significant at the $P<0.05$ level.

There was a close correlation between vessel density (200 × field) and peripheral uPA level ($r = 0.85$; $P<0.01$; 95% confidence interval 0.74–0.91). The correlation was still significant when the patients were separated into node-negative and node-positive groups (Table II).

The Ki-67 proliferation rates (23.9 ± 2.7%, $n = 42$) differ significantly in node-positive (28 ± 3%) and node-negative (19 ± 1.9%) patients ($P<0.05$). We have found a high correlation between the Ki-67 growth fraction and the uPA levels ($r = 0.91$, $P<0.001$) (Figure 4).

The macrophage grading did not differ significantly in node-positive (2.56 ± 0.21) and node-negative (2.05 ± 0.2) patients. In agreement with Müller *et al.* (1992), we found a ubiquitous distribution of macrophages and a preponderant

**Figure 3** PAI-1 is inversely related to steroid hormone receptors. PR, progesterone receptor.

localisation within the tumour stroma, but virtually no accumulation around hot spots of vascular proliferation. The macrophages were most frequent and in a high density at the margins of tumour cell islands. Sometimes they were localised within tumour islands (Müller *et al.*, 1992).

### Discussion

This study shows a significant correlation between the uPA and PAI-1 levels of breast cancer tissue extracts and the

**Figure 4** A high correlation between the Ki-67 growth fraction and the uPA levels exists.

**Figure 5** Correlation of uPA level and vessel count per 200 × field (vessel density); $n = 42$, $r = 0.85$, $P<0.05$.

density of microvessels and vascular invasion in histological sections (Figure 5). It is known that the first step in angiogenesis is a proteolytic degradation of vessel walls, and it is thought that this initiation of neovascularisation is triggered by collagenases, including type IV, and plasminogen activators released by endothelium cells (Mahadevan *et al.*, 1990).

Our results support the hypothesis that uPA and enzymes which disrupt vessel walls start their degradation, and thus neovascularisation, at the exterior vessel side after being released by tumour cells. The enzyme-caused vessel wall dissolution is a known stimulus which leads to a proliferation of endothelium cells, supported by various angiogenic factors, and subsequently to the formation of new blood vessels. Thus uPA and perhaps other proteolytic enzymes released by tumour cells may be involved in the initiation of angiogenesis; uPA may be an indirect angiogenic factor *in vivo*. Furthermore, the invasive chemotactic behaviour of endothelial cells at the tips of growing capillaries is facilitated by plasminogen activators and collagenases released by tumour cells as well as endothelium cells (Moscatelli *et al.*, 1981).

On the other hand, proliferating capillaries have fragmented basement membranes and are leaky, making them more penetrable by tumour cells than mature vessels (Nagy *et al.*, 1989). This may be an important factor which explains our result concerning the tumour vessel invasion. Tumours possessing high levels of uPA have 9% more vascular invasions than carcinomas with an uPA level lower than 3 ng $mg^{-1}$. This entry of tumour cells into circulation is the beginning of metastatic processes and may explain clinical investigations concerning uPA and breast cancer.

The fact that plasminogen activators are indispensable for tube formation of microvascular cells, and that they may induce angiogenesis *in vitro* (Yasunaga *et al.*, 1989; Sato *et al.*, 1993), strongly suggests a role for uPA in tumour neovas-

**Table III** Vessel density grade and macrophage grade

| *Grade* | *1* | *2* | *3* | *4* |
|---|---|---|---|---|
| Vessel density grade (*n*) | 10 | 17 | 9 | 6 |
| Percentage | 23.8 | 40.5 | 21.4 | 14.3 |
| Ki-67 (%) mean | 11.1 | 21.3 | 28 | 46.7 |
| uPA (ng $mg^{-1}$) mean | 1.1 | 3.5 | 5.4 | 9.9 |
| Macrophage grade (*n*) | 8 | 18 | 10 | 6 |
| Percentage | 19 | 42.8 | 23.8 | 14.3 |
| Ki-67 (%) mean | 11.5 | 19 | 30.2 | 44.8 |
| uPA (ng $mg^{-1}$) mean | 1.2 | 2.96 | 5.98 | 10.3 |

**Table II** Interesting correlations

| *Correlation* | r | r(s) | *95% CI* | P-*value* |
|---|---|---|---|---|
| uPA(p) *vs* vessel density (*n* = 42) | 0.85 | 0.92 | 0.74–0.91 | <0.01 |
| uPA *vs* vessel density (node positive) (*n* = 23) | 0.91 | 0.93 | 0.79–0.96 | <0.001 |
| uPA *vs* vessel density (node negative) (*n* = 19) | 0.84 | 0.97 | 0.62–0.94 | <0.001 |
| uPA (p) *vs* angio-invasion (*n* = 42) | 0.79 | 0.88 | 0.64–0.88 | <0.001 |
| PAI-1 *vs* uPA (*n* = 42) | 0.79 | 0.90 | 0.64–0.88 | <0.01 |
| PAI-1 *vs* vessel density | 0.74 | 0.86 | 0.56–0.85 | <0.01 |
| Vessel count (F VIII *vs* CD31) (*n* = 42) | 0.87 | 0.90 | 0.69–0.91 | <0.01 |
| Ki-67 *vs* uPA (*n* = 42) | 0.91 | 0.95 | 0.84–0.95 | <0.001 |
| ER *vs* PAI-1 | −0.42 | −0.85 | −0.69 to −0.12 | <0.05 |
| PR *vs* PAI-1 | −0.59 | −0.89 | −0.85 to −0.56 | <0.01 |

*r*, Pearson's correlation coefficient; *r*(s), Spearman's correlation coefficient; 95% CI, 95% confidence interval; ER, oestrogen receptor; PR, progesterone receptor.

cularisation. We postulate a close collaboration between tumour cells and tumour-associated macrophages in angiogenesis. The importance of macrophages in angiogenesis results from three qualities:

(1) Macrophages are resident in all breast carcinomas in larger numbers than other blood-borne cells, and distinct subtypes of macrophages can always be recruited from the bloodstream (Müller *et al*., 1992).
(2) They are functionally heterogeneous and can be activated from a quiescent non-angiogenic stage to an angiogenic stage (Assoian *et al*., 1987).
(3) Angiogenesis is a multistep process for which changes in the ECM have to be combined with the coordinated supply of appropriate angiogenic factors. Macrophages, and especially distinct subtypes of macrophages, are capable of providing several cytokines for the initiation, the maintenance and the termination of the angiogenic process.

**Figure 6** High uPA levels cause an increase in the percentage of vascular invasions. s.e.m. = standard error of the mean.

**Figure 7** Peripheral tumour area. Strong uPA reaction in close vicinity of a vessel. APAAP stain for uPA antigen (200 ×).

**Figure 8** Weak uPA reaction in a central tumour area. APAAP stain for uPA antigen (400 ×).

The connection between vessel density grade, macrophage grade, Ki-67 growth fraction and uPA level is represented in Table III. These results indirectly suggest a collaboration between macrophages and tumour cells in angiogenesis.

Recent studies have shown that elevated uPA and PAI levels in breast cancer tumour tissue correlate significantly with a shortened time until first recurrence and a shortened overall survival time in breast cancer patients, and that both enzymes are independent prognostic factors in node-negative breast cancer patients (Jänicke *et al*., 1990, 1993; Foekens *et al*., 1992). The essential findings in these studies were that breast cancer patients with either high uPA or high content of uPA inhibitor PAI-1 in their primary tumours have an increased risk of relapse and death. Our results partly confirm and explain these clinical investigations; high uPA levels are correlated with high vessel densities. This means that patients with high uPA levels have a greater vessel density and higher percentage of vascular invasions compared with patients who exhibit low uPA levels (Figure 6). Thus the tumour cell entry into circulation may happen more frequently at an early time, and it is this early tumour dissemination which leads to a shortened overall survival time.

An important result of this study is that central portions of breast carcinoma contain less uPA antigen than peripheral tumour parts. This was confirmed by the evaluation of histological sections stained for uPA antigen (Figures 7 and 8). This agrees with other authors who have found that in Lewis lung carcinoma uPA was located in the invading parts of the tumour, often in areas in which active infiltration and tissue destruction was taking place (Skriver *et al*., 1984).

This is in agreement with the fact that the areas of high neovascularisation were most frequent at the margins of the carcinoma. This heterogeneous distribution of uPA in breast cancer suggests that the tissue used for extraction should be taken at the tumour margins.

On the other hand, we did not find significant differences in uPA levels in central compared with peripheral tumour portions in carcinomas smaller than 1.5 cm. This may be explained by the difficulty in distinguishing between central and peripheral areas during tissue extraction and more likely the entire tumour tissue is the area of active infiltration and tissue destruction, so that in this confined group of small tumours the uPA content seems to be homogeneously distributed.

PAI-1 correlates weakly with uPA and with vessel density. It seems somewhat contradictory that the uPA inhibitor PAI-1 is also important for poor prognosis and that its ranking in multivariate analysis is close in order to that of uPA (Jänicke *et al*., 1991), since one would expect PAI-1 to act protectively by blocking the enzymatic activity of free and receptor-bound uPA. Jänicke *et al*. argued that excess release of PAI-1 might be of importance for reimplantation of circulating tumour cells, since formation of new stroma at the metastatic site requires the blockade of uPA-mediated degradation of extracellular matrix.

It was hypothesised that PAI-1 levels in tumour extracts may be a biochemical measure of the extent of neovascularisation (Grondahl-Hansen *et al*., 1993), since PAI-1 may play a role in angiogenesis (Montesano *et al*., 1990).

**Acknowledgements**
The authors would like to thank Mrs R Hannagarth and Mrs S Trochimczyk for performing the excellent immunohistochemistry. The technical assistance of Mr E Nacih is gratefully acknowledged. This study was supported by FA WAK Chemie, Bad Homburg Germany.

**References**

ASSOIAN RK, FLEURDELYS BE, STEVENSON HC, MILLER PJ, MADTES DK, RAINES EW, ROSS R AND SPORN MB. (1987). Expression and secretion of type beta transforming growth factor by activated human macrophages. *Proc. Natl Acad. Aci. USA*, **84**, 6020–6024.

BERSE B, BROWN LF, VAN DE WATER L, DVORAK HF AND SENGER DR. (1992). Vascular permeability factor (vascular endothelial growth factor) gene is expressed differentially in normal tissues, macrophages, and tumours. *Mol. Biol. Cell*, **3**, 211.

BROWN LF, DVORAK AM AND DVORAK HF. (1989). Leaky vessels, fibrin deposition and fibrosis: a sequence of events common to solid tumors and to many other types of disease. *Am. Rev. Respir. Dis.*, **140**, 1104–1107.

CORDELL IL, FALINI B, ERBER WN, GOSH AK, ABDULAZIZ Z, MACDONALD S, PULFORD KAF, STEIN H AND MASON DY. (1984). Immunoenzymatic labelling of monoclonal antibodies using immune complexes of alkaline phosphatase and monoclonal antialkaline phosphatase (APAAP complexes). *J. Histochem. Cytochem.*, **32**, 219–229.

DANO K, ANDREASEN PA, GRONDAHL-HANSEN J, KRISTENSEN P, NIELSEN LS AND SKRIVER L. (1985). Plasminogen activators. Tissue degradation and cancer. *Adv. Cancer Res.*, **44**, 139–266.

DVORAK HF. (1986). Tumors: wounds that do not heal. *N. Engl. J. Med.*, **315**, 161–165.

FALCONE DJ, MCCAFFREY TA, HAIMOVILZ-FRIEDMAN A AND GARCIA M. (1993*a*). Transforming growth factor-beta 1 stimulates macrophage urokinase expression and release of matrix-bound basic fibroblast grøwth factor. *J. Cell Physiol.*, **155**, 595–605.

FALCONE DJ, MCCAFFREY TA, HAIMOVILZ-FRIEDMAN A, VERGILIO J AND NICHOLSON AC. (1993*b*). Macrophage and foam cell release of matrix bound growth factors. Role of plasminogen activation. *J. Biol. Chem.*, **268**, 11951–11958.

FOEKENS J. (1992). Prognostic value of urokinase type plasminogen activator in 671 primary breast cancer patients. *Cancer Res.*, **52**, 6101–6105.

GELEHRTER TD, BAROUSKI-MILLER J, COLEMAN PL AND CWINKEL C. (1983). Plasminogen activator inhibitors and hormonal regulation in rat hepatoma cell line HTC. *Br. J. Mol. Cell Biochem.*, **53/54**, 11–21.

GOLDFARB RH AND LIOTTA LA. (1986). Proteolytic enzymes in cancer invasion and metastases. *Semin. Thromb. Hemostasis.*, **12**, 294–316.

GRONDAHL-HANSEN J, CHRISTENSEN IB AND ROSENQUIST C. (1993). High levels of urokinase type plasminogen activator (uPA) and its inhibitor PAI-1 in cytosolic extracts of breast carcinomas are associated with poor prognosis. *Cancer Res.*, **53**, 2513–2521.

JÄNICKE F, SCHMITT M, HAFTER R, HOLLRIEDER A, BABIE R, ULM K, GÖSSNER W AND GRAEFF H. (1990). Urokinase-type plasminogen activator (uPA) antigen is a predictor of early relapse in breast cancer. *Fibrinolysis*, **4**, 69–78.

JÄNICKE F, SCHMITT M AND GRAEFF H. (1991). Clinical relevance of the urokinase-type and tissue-type plasminogen activators and of their inhibitor PAI-1 in breast cancer. *Semin. Thromb. Hemostasis*, **17**, 303–312.

JÄNICKE F, SCHMITT, PACHE L, ULM K, HARBECK N, HÖFLER H AND GRAEFF H. (1993). Urokinase (uPA) and its inhibitor PAI-1 are strong and independent prognostic factors in node negative breast cancer. *Breast Cancer Res. Treat.*, **24**, 195–208.

KOBAYASHI H, SCHMITT M, GORETZKI L, CHUCHOLOWSKI N, CALRETE J, KRAMER M, GÜNZLER WA, JÄNICKE F AND GRAEFF H. (1990). Cathepsin B efficiently activates the soluble and the tumor cell receptor bound form of the proenzyme urokinase type plasminogen activator (pro-uPA). *J. Biol. Chem.*, **266**, 5147–5152.

LE DOUSSAL V, TUBIANA-HULIN M, FRIEDMAN S, HACENE K, SPYRATOS F AND BRUNTE M. (1989). Prognostic value of the histologic grade nuclear components of Scarff Bloom Richardson (SBR): an improved score modification based on a multivariate analysis of 1262 invasive ductal breast carcinomas. *Cancer*, **64**, 1914–1921.

LIU MM, WANG DL AND LIU CY. (1990). Interactions between fibrin, collagen and endothelial cells in angiogenesis. *Adv. Exp. Med. Biol.*, **281**, 319–331.

MAHADEVAN V AND HART JR. (1990). Metastases and angiogenesis. *Acta Oncol.*, **29**, 97–103.

MILES A AND PLOW EF. (1988). Plasminogen receptors: Ubiquitous sites for cellular regulation of fibrinolysis. *Fibrinolysis*, **2**, 61–71.

MONTESANO R, PEPPER MS AND MOHLESTEINLEIN U. (1990). Increased proteolytic activity is responsible for the aberrant morphogenetic behaviour of endothelial cells expressing the middle T-oncogene. *Cell*, **62**, 435–445.

MOSCATELLI D, GROSS JL AND RIFKIN DB. (1981). Angiogenic factors stimulate plasminogen activator and collagenase production by capillary endothelium cells. *J. Cell. Biol.*, **91**, 201a.

MÜLLER H. (1992). *The Human Mammary Carcinoma*. Gustav Fischer: Stuttgart.

NAGY JA, BROWN LF AND SENGER DR. (1989). Pathogenesis of tumor stroma generation: a critical role for leaky blood vessels and fibrin deposition. *Biochim. Biophys. Acta*, **948**, 305–326.

SATO Y, OKAMURA K, MORIMOTO A, HAMANAKA R, HAMAGUCHI K, SHIMADA T, ONO M, KOHNO K, JAKATA T AND KUWANO M. (1993). Indispensable role of tissue-type plasminogen activator in growth factor-dependent tube formation of human microvascular endothelial cells in vitro. *Exp. Cell Res.*, **204**, 223–229.

SCHMITT M, ANAMAYA N, HENSCHEN A, HOLLRIEDER A, HAFTER R, GULBA D, JÄNICKE F AND GRAEFF H. (1989). Elastase released from human granulocytes stimulated with N-formyl-chemotactic peptide prevents activation of tumor cell prourokinase. *FEBS. Lett.*, **255**, 83–88.

SKRIVER L, LARSON LJ, KIELBERG V, NIELSEN LS, ANDREASEN PB, KRISTENSEN P AND DANO K. (1984). Immunocytochemical localisation of urokinase-type plasminogen activator in Lewis lung carcinoma. *J. Cell Biol.*, **99**, 753–758.

STUMP D. (1986). Purification and characterisation of low molecular weight form of single-chain urokinase-type plasminogen activator. *J. Biol. Chem.*, **261**, 17120–17126.

THOMPSON WD, SMITH EB, STIRK CM, MARSHALL FI, STOUT AJ AND KOCCHAR A. (1992). Angiogenic activity of fibrin degradation products is located in fibrin fragment. *Eur. J. Pathol.*, **168**, 47–57.

VAN NETTEN JP, ASHMEAD BJ, PARKER IG, THORNTON C, FLETCHER D, CAVERS P AND BRIGDEN ML. (1993). Macrophage-tumor cell associations, a factor in metastasis of breast cancer? *J. Leu. Biol.*, **54**, 360–362.

VASALLI JD, BACCINO D AND BELIN D. (1985). A cellular binding site for the Mr 55000 form of the human plasminogen activator urokinase. *J. Cell Biol.*, **100**, 86–92.

WEST DC, HAPSON IN, ARNOLD F AND KUMAR. (1985). Angiogenesis induced by degradation products of hyaluronic acid. *Science*, **228**, 1324–1326.

WEST DC AND KUMAR S. (1989). The effect of hyaluronate and its oligosaccharides on endothelial cell proliferation and monolayer integrity. *Exp. Cell Res.*, **183**, 179–196.

YASUNAGA C, NAKASHIMA Y AND SUEISHI K. (1989). A role of fibrinolytic activity in angiogenesis. Quantitative assay using in vitro method. *Lab. Invest.*, **61**, 698–704.